# Diabetes and baseline glucose are associated with inflammation, left ventricular function and short- and long-term outcome in acute coronary syndromes: role of the novel biomarker Cyr 61

**DOI:** 10.1186/s12933-019-0946-6

**Published:** 2019-10-31

**Authors:** Patric Winzap, Allan Davies, Roland Klingenberg, Slayman Obeid, Marco Roffi, François Mach, Lorenz Räber, Stephan Windecker, Christian Templin, Fabian Nietlispach, David Nanchen, Baris Gencer, Olivier Muller, Christian M. Matter, Arnold von Eckardstein, Thomas F. Lüscher

**Affiliations:** 1 Center for Molecular Cardiology, University of Zurich, Zurich, Switzerland; 2 Royal Brompton and Harefield Hospitals and Imperial College, Sydney Street, London, SW3 6NP UK; 3 Department of Cardiology, Kerckhoff Heart and Thorax Center, Bad Nauheim, Germany; 4 Department of Cardiology, University Heart Center Zurich, University Hospital Zurich, Zurich, Switzerland; 5 Department of Cardiology, Hopital Universitaire de Geneve, Geneva, Switzerland; 6 Department of Cardiology, Swiss Heart Centre, Inselspital, Bern, Switzerland; 7 Center for Primary Care and Public Health (Unisanté), University of Lausanne, Lausanne, Switzerland; 8 Service of Cardiology, Centre Hospitalier Universitaire Vaudois, Lausanne, Switzerland; 9 Institute of Clinical Chemistry, University Hospital Zurich, Zurich, Switzerland

**Keywords:** Acute coronary syndromes, Diabetes, Glucose, Inflammation, Major cardiovascular and cerebrovascular events, Mortality

## Abstract

**Background:**

Hyperglycemia in the setting of an acute coronary syndrome (ACS) impacts short term outcomes, but little is known about longer term effects. We therefore designed this study to firstly determine the association between hyperglycemia and short term and longer term outcomes in patients presenting with ACS and secondly evaluate the prognostic role of diabetes, body mass index (BMI) and the novel biomarker Cyr61 on outcomes.

**Methods:**

The prospective Special Program University Medicine-Acute Coronary Syndrome (SPUM-ACS) cohort enrolled 2168 patients with ACS between December 2009 and October 2012, of which 2034 underwent PCI (93.8%). Patients were followed up for 12 months. Events were independently adjudicated by three experienced cardiologists. Participants were recruited from four tertiary hospitals in Switzerland: Zurich, Geneva, Lausanne and Bern. Participants presenting with acute coronary syndromes and who underwent coronary angiography were included in the analysis. Patients were grouped according to history of diabetes (or HbA1c greater than 6%), baseline blood sugar level (BSL; < 6, 6–11.1 and > 11.1 mmol/L) and body mass index (BMI). The primary outcome was major adverse cardiac events (MACE) which was a composite of myocardial infarction, stroke and all-cause death. Secondary outcomes included the individual components of the primary endpoint, revascularisations, bleeding events (BARC classification) and cerebrovascular events (ischaemic or haemorrhagic stroke or TIA).

**Results:**

Patients with hyperglycemia, i.e. BSL ≥ 11.1 mmol/L, had higher levels of C-reactive protein (CRP), white blood cell count (WBC), creatinine kinase (CK), higher heart rates and lower left ventricular ejection fraction (LVEF) and increased N-terminal pro-brain natriuretic peptide. At 30 days and 12 months, those with BSL ≥ 11.1 mmol/L had more MACE and death compared to those with BSL < 6.0 mmol/L or 6.0–11.1 mmol/L (HR-ratio 4.78 and 6.6; p < 0.001). The novel biomarker Cyr61 strongly associated with high BSL and STEMI and was independently associated with 1 year outcomes (HR 2.22; 95% CI 1.33–3.72; Tertile 3 vs. Tertile 1).

**Conclusions and relevance:**

In this large, prospective, independently adjudicated cohort of in all comers ACS patients undergoing PCI, both a history of diabetes and elevated entry glucose was associated with inflammation and increased risk of MACE both at short and long-term. The mediators might involve increased sympathetic activation, inflammation and ischemia as reflected by elevated Cyr61 levels leading to larger levels of troponin and lower LVEF.

*Trial registration* Clinical Trial Registration Number: NCT01000701. Registered October 23, 2009

## Background

Cardiovascular disease (CVD) remains the main cause of deaths worldwide. Indeed, in 2008 more than 17 million people died from CVDs and 10% of the global disease burden has been attributed to CVD [1]. The most common forms are chronic and acute coronary syndromes (ACS). Their causes are multifactorial, but involve behavioural risk factors such as physical inactivity, unhealthy diets, alcohol abuse and weight gain [2] which are also leading to the metabolic syndrome and diabetes. Elevated blood glucose levels are major determinants of atherosclerosis and plaque formation and unfavourable outcomes in the population at large [3].

The global prevalence of diabetes in adults aged over 25 is 10%. According to the *International Diabetes Foundation* 425 million are estimated to have diabetes in 2017 and if this trend continues, there will be 642 million adult diabetics in 2040 [4]. More than half of them are expected to die from CVD. Indeed, cardiovascular events are two to four times more common in individuals with type 1 or type 2 diabetes [5]. Of note, the risk of developing type 2 diabetes, coronary heart disease and/or ischemic stroke increases steadily with an increasing BMI [6–10].

Hyperglycemia is associated with unfavourable short term outcomes in both diabetics and non-diabetics presenting with ACS [11, 12] and also in those with cardiogenic shock [13]. However, the associations involved between hyperglycemia and short as well as long-term outcomes are not well understood. We investigated the relationship between hyperglycemia and possible mechanisms reflected by established and novel biomarkers of both short- and long-term outcomes in patients presenting with ACS undergoing PCI. Furthermore, we analysed the impact of the diagnosis of diabetes, and the relationship between diabetes and body mass index (BMI) on outcome.

## Methods

### Study population

The prospective multi-centre *Special Program University Medicine* (SPUM)-ACS Biomarker cohort (ClinicalTrials.gov Nr. NCT01000701) recruited patients who were referred for coronary angiography with the primary diagnosis of ACS to one of four Swiss University Hospitals (Zurich, Bern, Lausanne and Geneva) between December 2009 and October 2012. It comprised consecutive recruitment and follow-up performed at 30 days (phone call) and 1 year (clinical visit). Female and male patients aged 18 years or older presenting within 5 days (preferably within 72 h) after pain onset with the main diagnosis of ST-elevation myocardial infarction (STEMI), non-ST-elevation myocardial infarction (NSTEMI) or unstable angina (UA) were included. Within this consortium, a centralised electronic database was implemented providing comprehensive information on all patients. All adverse events occurring within 1 year after the index ACS event were ascertained at 30 days (telephone visit) and 1 year (clinical visit) and adjudicated by an independent adjudication committee consisting of 3 experienced cardiologists.

### Patient selection

Patients had symptoms compatible with angina pectoris (chest pain, dyspnea) and fulfilled at least one of the following criteria: (a) ECG changes such as persistent ST-segment elevation or depression, T-inversion or dynamic ECG changes, new left bundle branch block (LBBB); (b) evidence of positive (predominantly conventional) troponin by local laboratory reference values; (c) known coronary artery disease, specified as status after myocardial infarction, or PCI or newly documented ≥ 50% stenosis of an epicardial coronary artery during the initial catheterisation. Exclusion criteria included severe physical disability, inability to comprehend the study or life expectancy of less than 1 year for non-cardiac reasons.

### Measurement of biomarkers

Blood was drawn from the radial or femoral sheath at the beginning of the procedure and, following centrifuge, was stored at − 80 °C to allow for later analysis. Plasma glucose was measured using local laboratories with blood collected at the time of presentation to the hospital, therefore patients were not necessarily fasting prior to blood collection. Biomarkers were measured in serum blinded to the current analysis in a central core laboratory (AvE, Department of Clinical Chemistry, University Hospital Zurich, Switzerland). C-reactive protein (CRP), high-sensitivity troponin T (hsTnT), N-terminal pro-brain natriuretic peptide (NT-proBNP) were measured using immunoassays and the COBAS8000 autoanalyser from Roche Diagnostics, Mannheim, Germany). In addition, the soluble matricellular protein Cysteine-rich angiogenic inducer (Cyr61, syn. CCN1) was determined in serum using a immunoassay (EIA-5108, DRG Instruments GmbH, Marburg, Germany) [14].

### Definitions and endpoints

The primary endpoint of major adverse cardiovascular events (MACE) was defined as the composite of death, myocardial infarction (MI) and stroke. Secondary endpoints included the individual components of the primary endpoint, revascularisations, bleeding events (BARC classification) and cerebrovascular events (ischaemic or haemorrhagic stroke or TIA). Diabetes was defined as patients with a clinically known history of diabetes and/or on oral or injectable antidiabetic medication and/or HbA1c > 6.5% on admission. In patients with measured HbA1c, diabetes was considered present in individuals with HbA1c > 6.5% and ruled-out if HbA1c was < 5.7%. Pre-diabetes was defined as a HbA1c between 5.7 and 6.5%.

### Data analysis and statistics

To compare diabetics with non-diabetics we used two-sided t-tests to compare two groups assuming equal variances and ANOVA to compare more than two groups. If assumptions of normality were violated, rank sum test was used to compare two groups and Kruskal–Wallis tests were used to compare more than two groups with an ordinal classification (e.g. blood glucose level). Significance levels were chosen at p < 0.05. Patients were stratified according to BSL levels on presentation, BMI categories and medication history (insulin; Oral hypoglycemic agents and diabetics without specific antidiabetic medication).

Outcomes after ACS with PCI were compared between diabetics and non-diabetics at 30 days and 1 year using Kaplan–Meier survival curves and evaluated using log rank tests. Outcomes were also compared in patients with entry glucose of < 6.0 mmol/L, 6.0–11.1 mmol/L and > 11.1 mmol/L and comparison was made using log rank tests. To assess more closely the relationship between extreme hyperglycemia (glucose > 11.1 mmol/L) and major adverse cardiac events (independent of diabetic status), we performed Cox regression analysis adjusting for patients diabetic status using an entry glucose of < 6.0 mmol/L as a reference. We used landmark analyses to evaluate the unadjusted impact of hyperglycemia in survivors at 30 days to 365 days. Log rank tests was used to compare groups in the landmark analysis.

For the analysis of body mass index (BMI) on outcomes after ACS, patients were grouped according to their body mass index (BMI): BMI < 25 kg/m^2^ (normal), BMI 25–29.9 kg/m^2^ (overweight) and BMI > 30 kg/m^2^ (obese) and outcomes were compared using Kaplan–Meier survival analyses and log rank tests. Multivariate logistic regression was used to assess the relationship between above and below median Cyr61 levels and hyperglycemia. Cox regression analysis was performed to then assess the relationship between tertiles of Cyr61 levels and outcomes, taking into account potential confounders such as MI type, leucocyte count and hyperglycemia.

## Results

### Baseline characteristics

#### Baseline demographics

Out of 2168 patients with an ACS referred for coronary angiography, 2034 underwent PCI (93.8%) which were the basis for the current analysis. Overall, 82% of patients were male and 18% were female. Out of these 2034 ACS patients, 18.3% (n = 373) were diabetics. The male–female ratio was similar among diabetic and non-diabetic patients (Table 1). However, diabetic patients were older (p < 0.001) and had a higher BMI compared to non-diabetics patients (p < 0.001). A higher proportion of diabetics patients had a BMI > 30 kg/m^2^ (33.4% vs. 19.1%; p < 0.001). Diabetic patients also had a higher heart rate (78.4 bpm vs. 75.5 bpm; p = 0.001), but no significant difference in blood pressure compared to non-diabetics. Furthermore, left ventricular ejection fraction (LVEF) was lower in diabetic patients compared to non-diabetics (p < 0.01, Table 1).

**Table 1:** Demographics, hemodynamic parameters and major laboratory values in non diabetics (NO DM) and diabetics (DM)

	No DM (N = 1661)	DM (N = 373)	p value
BMI	26.8 ± 4.1	28.8 ± 4.7	< 0.001
Sex			
♂	1313	295	0.99
♀	348	78	
% female	21.0%	20.9%	0.91
Age (years)	62.8 ± 12.4	66.5 ± 12.2	< 0.001
Hypertension	883 (53%)	291 (78%)	< 0.001
Previous MI	198 (11.9%)	83 (22.2%)	< 0.001
Previous PCI	244 (14.7%)	92 (24.7%)	< 0.001
Previous CABG	62 (3.7%)	36 (9.7%)	< 0.001
Previous stroke	29 (1.7%)	18 (4.8%)	< 0.001
Previous TIA	18 (1.1%)	15 (4.0%)	< 0.001
ACS type			
STEMI	948 (57.1%	174 (46.7%)	< 0.001
NSTEMI	663 (39.9%)	180 (48.2%)	
UA	50 (3.0%)	19 (5.1%)	
LVEF (%)	51.7 ± 11.2	48.9 ± 12.3	< 0.001
Heart rate (BPM)	75.5 ± 16.0	78.4 ± 15.7	0.001
Systolic BP (mmHg)	130.1 ± 23.5	131.9 ± 23.1	0.18
Diastolic BP (mmHg)	75.7 ± 14.5	75.4 ± 16.5	0.70
HDL-c (mmol/L)	1.2 ± 0.3	1.1 ± 0.3	< 0.001
LDL-c (mmol/L)	3.3 ± 1.1	2.7 ± 1.2	< 0.001
Triglycerides (mmol/L)	1.005 (0.66–1.53)	1.14 (0.81–1.83)	< 0.001
Cholesterol (mmol/L)	5.0 ± 1.2	4.5 ± 1.4	< 0.001
Leukocytes (g/L)	9.7 (7.52–12.2)	9.34 (7.4–12.1)	0.197
CRP (mg/L)	2.5 (1.1–7)	3.9 (1.6–11.1)	< 0.001
Glucose (mmol/L)	6.6 ± 1.9	9.8 ± 4.6	< 0.001
HBa1c (%)	5.8 ± 0.3	7.6 ± 1.9	< 0.001
hs troponin T (µg/L)	0.20 (0.06–0.71)	0.19 (0.057–0.681)	0.49
Creatine kinase (U/L)	235 (114–539)	185 (93–419)	< 0.001
nt-probnp (ng/L)	337 (113–1155)	633 (192–2325)	< 0.001
Cyr61 (pg/mL)	510 (340–951)	487 (344–780)	0.25

*BMI* body mass index, *CRP* C-reactive protein, *HBA1C* haemoglobin A1c, *HDL-C* high density lipoprotein cholesterol, *LDL-C* low density lipoprotein cholesterol, *LVEF* left ventricular ejection fraction, *STEMI* ST segment elevation myocardial infarction, *NSTEMI* non ST-segment elevation myocardial infarction, *UA* unstable angina

#### Laboratory values and biomarkers

Diabetic patients had significantly lower HDL-cholesterol (HDL-C), lower LDL-cholesterol (LDL-C) and lower total cholesterol, but elevated triglycerides compared to non-diabetics (all p < 0.001). In addition, diabetic patients had significantly higher levels of CRP (p < 0.001), while leucocyte counts were similar (Table 1).

#### Glucose levels at presentation

**Fig. 1:**
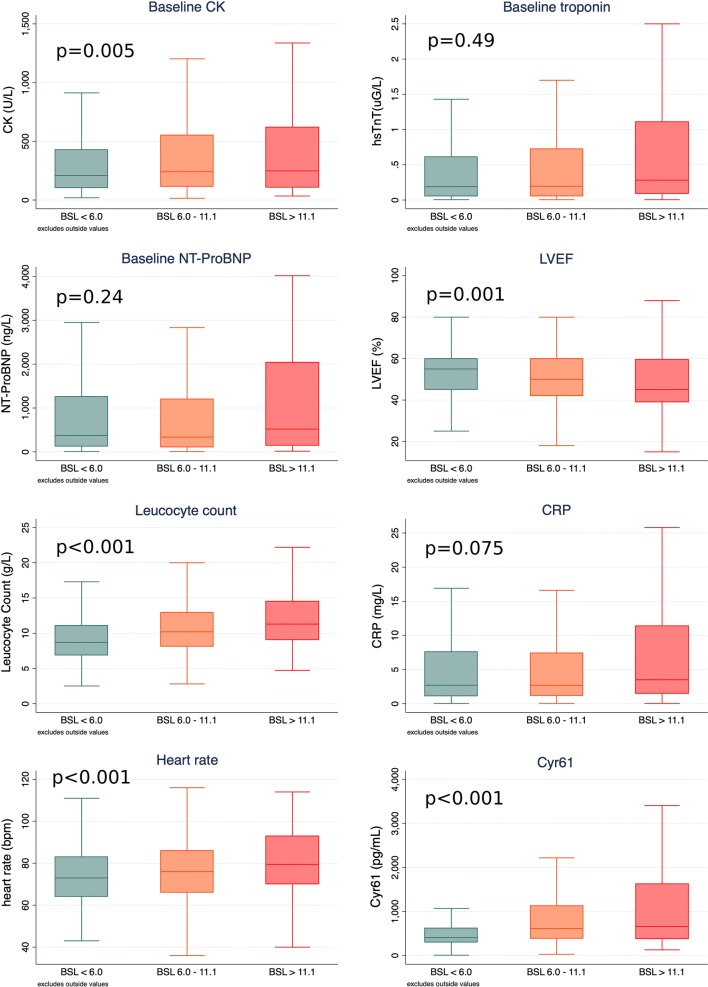
Box plots of selected variables and biomarkers according to glucose level at baseline. Differences between groups analysed using either ANOVA or Kruskal–Wallis tests

The median glucose on arrival was 8.3 mmol/L in diabetics and 6.1 mmol/L in non-diabetics, and 7.4% (n = 134) of patients presented with glucose ≥ 11.1 mmol/L (Fig. 1). In patients with a history of diabetes or with HbA1c > 6.5%, 28.5% (n = 97) had glucose ≥ 11.1 mmol/L, compared to only 2.5% (n = 37) of patients with previously unknown diabetes. There were significant differences in clinical parameters between these glucose groups, with patients with higher glucose having a higher heart rate, higher levels of CRP, higher leucocyte count and NT-proBNP and lower LVEF (Additional file 1: Table S1). Nearly all NSTEMI patients with glucose ≥ 11.1 mmol/L had a diagnosis of diabetes (29/30; 96.7%) whereas only two-thirds of STEMI patients with glucose ≥ 11.1 mmol/L had a diagnosis of diabetes (62/92; 67.4%).

### Clinical outcomes in diabetics and non-diabetics

#### Diabetics versus non-diabetics

**Fig. 2:**
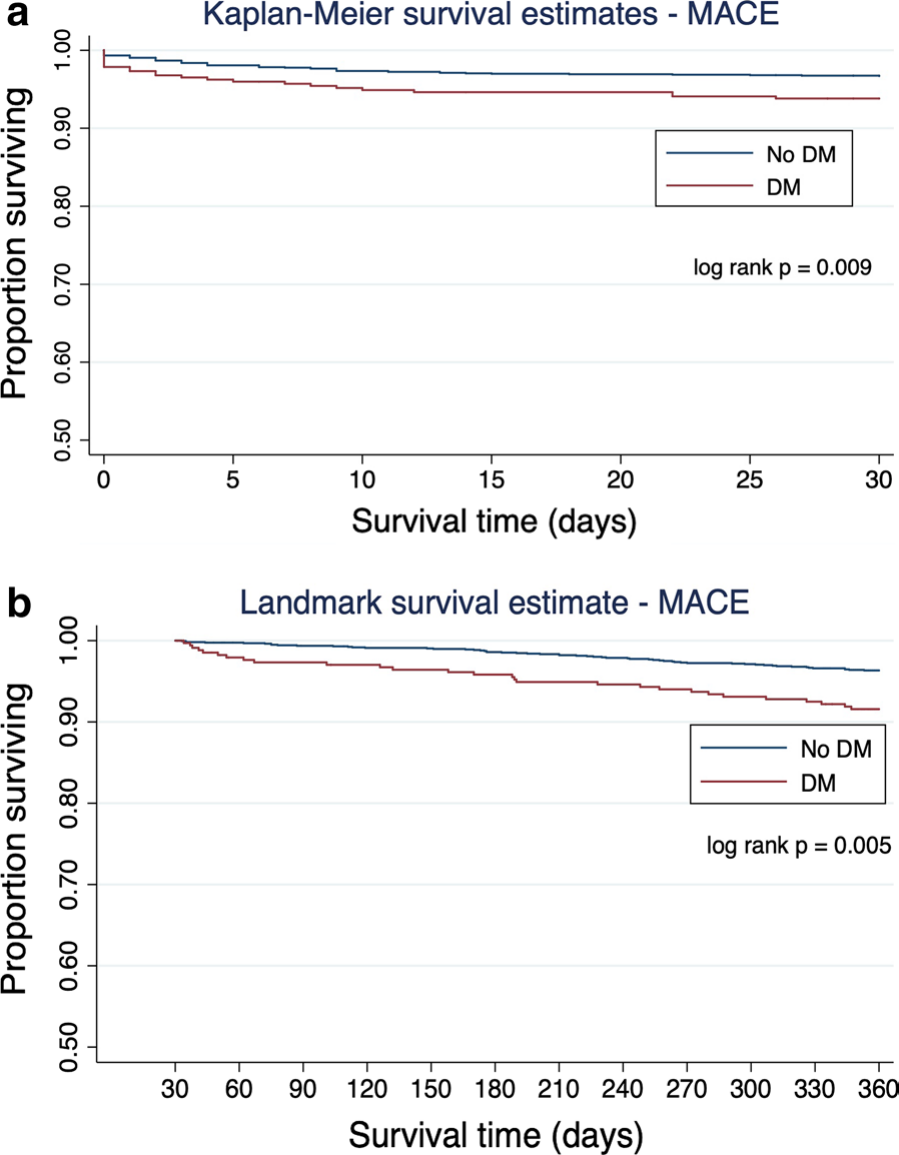
**a** Kaplan–Meier curves of major adverse cardiovascular events (MACE) in diabetics versus non-diabetics in the first 30 days. **b** Landmark survival curves (30 days to 1 year) of major adverse cardiovascular events in diabetics versus non-diabetics

**Fig. 3:**
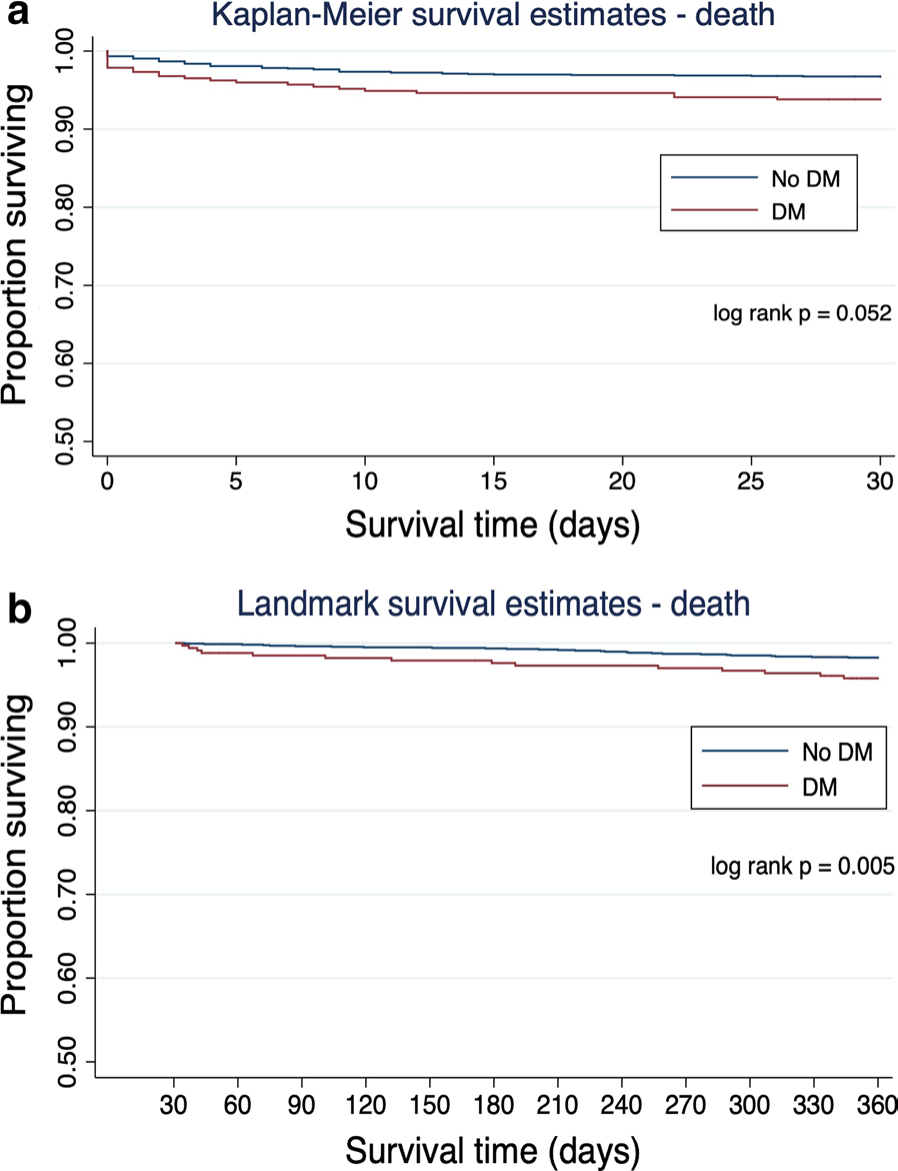
**a** Kaplan–Meier survival curves of mortality in diabetics versus non-diabetics in the first 30 days. **b** Landmark survival analysis using Kaplan–Meier survival curves to assess mortality in diabetics versus non-diabetics from 30 to 365 days

At 30 days, 6.2% (n = 23) of diabetic patients had experienced the primary endpoint compared with 3.3% (n = 55) of non-diabetics (p < 0.009; Fig. 2a). At 1 year, 13.7% (n = 51) of diabetics had experienced the primary endpoint compared to 6.7% (n = 112) of non-diabetics (p < 0.001). Thirty-day mortality was 3.2% (n = 12) in diabetics and 1.7% (n = 28) in non-diabetics (p = 0.054; Fig. 3a). This trend towards a higher mortality in diabetics reached statistical significance at 1 year (7.0% vs. 3.4%; p = 0.002; Fig. 3).

On landmark analysis, the diagnosis of diabetes was associated with the primary endpoint at 1 year in survivors to 30 days (log rank test p = 0.005; Fig. 2b). Furthermore, the diagnosis of diabetes was associated with increased risk of death at 1 year in those who survived to 30 days (log rank test p = 0.005; Fig. 3b).

There was a significantly higher stroke rate in diabetics compared to non-diabetics at 30 days (1.9% vs. 0.4%; p = 0.001) and 1 year (2.7% vs. 0.8%; p = 0.002). However, revascularisation rates did not differ between diabetics and non-diabetics (2.1% vs. 1.7%; p = 0.602) at 30 days and 1 year (7.8% vs. 5.9%; p = 0.176). Also, there was no significant difference in bleeding in diabetics and non-diabetics at 30 days (4.0% vs. 4.9%; p = 0.482) or 1 year (5.9% vs. 7.8%; p = 0.201).

### Outcomes according to baseline glucose

**Fig. 4:**
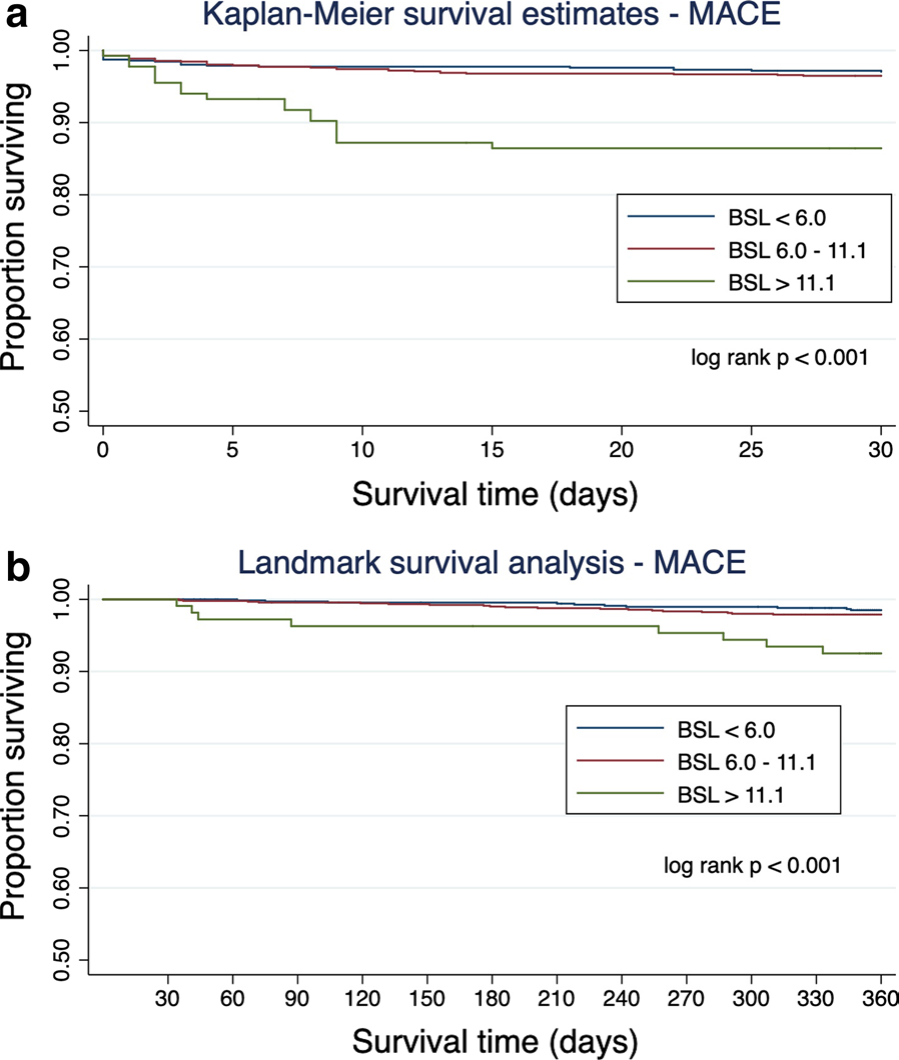
**a** Kaplan–Meier curves of MACE in ACS patients within 30 days according to plasma glucose levels on presentation. **b** Landmark survival analysis using Kaplan–Meier survival curves comparing outcomes from 30 days to 1 year according to plasma glucose levels on presentation

At 30 days, patients with glucose upon presentation of ≥ 11.1 mmol/L had significantly higher risk of the primary endpoint and mortality (Fig. 4). This was confirmed on univariate Cox regression analysis where a BSL ≥ 11.1 mmol/L was associated with a hazard ratio of 4.78 (95% CI 2.55–8.97; p < 0.001) for the primary endpoint at 30 days and a hazard ratio of 6.6 (95% CI 3.65–11.9; p < 0.001) for death at 30 days compared to patients with BSL < 6.0 mmol/L. After adjustment for diabetes history, the relationship between hyperglycemia and MACE remained significant with a hazard ratio of 5.21 (95% CI 2.47–10.98; p < 0.001). The hazard ratio for patients with a glucose of 6.0–11.1 mmol/L was 1.21 (95% CI 0.69–2.10).

Using landmark analysis, including only survivors at 30 days, patients with glucose ≥ 11.1 mmol/L still had significantly higher risk of MACE from 30 to 365 days (log rank p = 0.005; Fig. 4b). The rate of stroke at 30 days was significantly different between groups and was 2.24% in those with glucose ≥ 11.1 mmol/L, 0.93% with glucose 6–11.1 mmol/L and 0.14% in glucose < 6 mmol/L (p = 0.016). There was no difference between groups at either 30 days or 1 year in terms of revascularisation or bleeding.

When stratifying by infarct type (STEMI vs. NSTEMI/Unstable Angina), hyperglycemia (BSL ≥ 11.1 vs. BSL < 6.0) was predictive of outcomes at 365 days in both groups. The impact was more pronounced for STEMI patients (HR 5.64; 95% CI 2.95–10.76) compared to NSTEMI/UA patients (HR 3.02; 95% CI 1.25–7.31).

Finally, we found that the impact of hyperglycemia on outcomes was more pronounced in non-diabetic patients compared to diabetics. Non-diabetics presenting with glucose ≥ 11.1 mmol/L had a 30 day MACE rate of 21.6% (p < 0.001 compared to glucose < 6 mmol/L and 6–11.1 mmol/L), while diabetics presenting with glucose ≥ 11.1 mmol/L had a 30 day MACE rate of 10.3% (p = 0.06 compared to glucose < 6 mmol/L and 6–11.1 mmol/L). At 1 year non-diabetics presenting with glucose ≥ 11.1 mmol/L had a MACE rate of 27% (p < 0.001 compared to glucose < 6 mmol/L and 6–11.1 mmol/L), while diabetics presenting with glucose ≥ 11.1 mmol/L had a MACE rate of 21.7% (p = 0.01 compared to glucose < 6 mmol/L and 6–11.1 mmol/L).

### Association between Cyr61, diabetes, hyperglycemia and outcomes

**Fig. 5:**
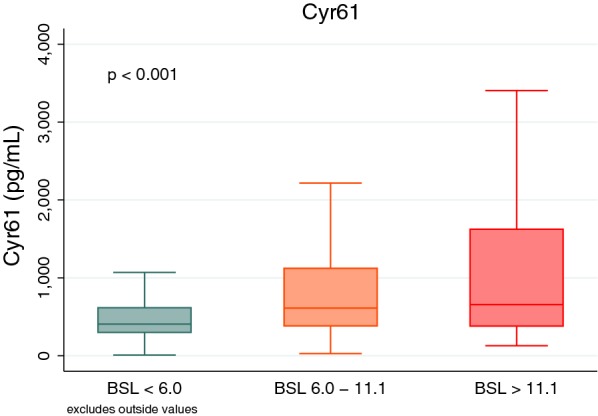
Box plot demonstrating change in Cyr61 levels as glucose increases, stratified by infarct type (STEMI vs. NSTEMI/UA)

In patients with glucose ≥ 11.1 mmol/L, levels of Cyr61 were significantly higher than those with glucose levels < 6.0 mmol/L or 6.0 mmol/L to 11.1 mmol/L (p < 0.001; Fig. 1). Furthermore levels of Cyr61 were higher across all categories of glucose for patients with STEMI while Cyr61 levels did not change significantly across glucose groups in NSTEMI/unstable angina (Fig. 5). Although both Cyr61, CRP and leucocyte count levels were higher in patients with glucose ≥ 11.1 mmol/L (Fig. 1), there was a weak negative correlation between Cyr61 and CRP (r = − 0.15; p < 0.001) levels, while a moderately positive correlation existed between Cyr61 and leucocyte count (r = 0.26; p < 0.001). There was no difference in Cyr61 levels in patients with HbA1c ≥ 8.0% compared to those with HbA1c < 8.0% (p = 0.13). On univariate analysis, patients with BSL > 11.1 mmol/L had significantly higher odds of having Cyr61 levels above the median (OR 2.79; 95% CI 1.87–4.16; p < 0.001). Given the known association between higher Cyr61 levels and STEMI [14], we adjusted for STEMI and other risk factors including age, gender, BMI, hypertension, diabetes, LDL cholesterol, LVEF, troponin T, STEMI and serum creatinine and CRP. There was still a strong relationship between hyperglycemia (as defined by BSL ≥ 11.1 mmol/L) and above median Cyr61 levels (OR 2.45; 95% CI 1.16–5.15, p = 0.018).

In terms of outcomes, we grouped Cyr61 levels into tertiles and found at 30 days using univariate cox regression analysis, a significantly higher risk of MACE in patients in the highest Cyr61 tertile (HR 2.39; 95% CI 1.28–4.47). After adjustment for STEMI, leucocyte count and glucose group, the relationship was not significant (HR 1.85; 95% CI 0.88–3.88) while there was a significant relationship between BSL ≥ 11.1 mmol/L and MACE (HR 5.55; 95% CI 2.71–11.37). At 1 year, after performing the same adjustments, there was a significant relationship between outcomes and the highest Cyr61 tertile (HR 2.22; 95% CI 1.33–3.72) as well as a persistent relationship between BSL ≥ 11.1 mmol/L and outcomes (HR 3.92; 95% CI 2.29–6.72).

### Outcomes and BMI

Outcomes according to BMI were analysed in 2’004 patients according to 3 different classes of BMI levels. There were significant differences among BMI groups at baseline. A significantly higher proportion of diabetics (34.4% vs. 19.2%; p < 0.001) had BMI > 30 kg/m^2^. Patients with BMI > 30 had the highest systolic (133.9 ± 23.2 mmHg) and diastolic (78.2 ± 15.1 mmHg) blood pressure in comparison to those with BMI 25–29.9 (systolic 130.2 ± 23.3, diastolic 75.9 ± 14.7 mmHg; p < 0.01) or a BMI < 25 (128.6 ± 23.2/73.9 ± 14.7 mmHg; p < 0.001). Moreover, the group with BMI > 30 showed the highest heart rate (77.4 ± 15.2 bpm; p < 0.003 vs. BMI 25–29.9, p = 0.52 vs. BMI < 25), higher CRP levels (12.5 ± 25.1 mg/L; p < 0.03 vs. BMI 25–29.9, p < 0.07 vs. BMI < 25) and the highest entry glucose levels (7.5 ± 3.1 mmol/L; p < 0.001 vs. BMI < 25, p < 0.05 vs. BMI 25–29.9) in comparison with the other groups.

There were no significant differences in the primary endpoint at 1 year between the three BMI groups (p = 0.211) (Table 2). When patients were grouped according to their history of diabetes, there was a significantly higher risk of the primary endpoint in diabetic patients with a BMI < 25 kg/m^2^ compared to diabetic patients with BMI 25–29.9 kg/m^2^ and BMI >=30 kg/m^2^ (Table 2). In terms of 1-year mortality, diabetic patients with a BMI < 25 kg/m^2^ had the highest mortality (14.3%), which was significantly different compared to the other BMI groups. There was no difference in outcomes in non-diabetics stratified by BMI groups (p = 0.065) (Table 2).

**Table 2:** Body mass index (BMI) and outcomes in diabetics and non-diabetics

	BMI < 25	BMI 25–29.9	BMI > 30	p value
MACE (1 year)				
Diabetics	21/91 (23.1%)	16/178 (9.0%)	16/135 (14.1%)	0.001
Non diabetics	36/550 (6.6%)	44/744 (5.9%)	15/306 (4.9%)	0.621
Death (1 year)				
Diabetics	13/91 (14.3%)	9/178 (5.1%)	9/135 (6.7%)	0.023
Non-diabetics	21/550 (3.8%)	17/744 (2.3%)	4/306 (1.3%)	0.065
Revascularization (1 year)				
Diabetics	13/91 (14.3%)	11/178 (6.2%)	7/135 (5.2%)	0.025
Non-diabetics	26/550 (4.7%)	53/744 (7.1%)	15/306 (4.9%)	0.14
Stroke (1 year)				
Diabetics	1/91 (1.1%)	5/178 (2.8%)	3/135 (2.2%)	0.667
Non-diabetics	5/550 (0.9%)	5/744 (0.7%)	3/306 (1.0%)	0.838

## Discussion

This large prospective real-world ACS study shows that in both diabetic and non-diabetic patients undergoing primary PCI (1) hyperglycemia on admission of ≥ 11.1 mmol/L, but not between 6.0 and 11.1 mmol/L is associated with inflammation, increased heart rate, larger troponin levels and reduced LVEF; (2) even with current guideline-based management hyperglycemia remains a powerful risk factor for 30 day and 1 year outcomes (particularly in non-diabetics) (4) In diabetics, MACE including stroke and mortality was higher than in non-diabetics and (5) the novel inflammation and myocardial injury biomarker Cyr61 [14] was most strikingly associated with hyperglycemia, STEMI and was predictive of outcomes.

### Hyperglycemia outcomes and potential mechanisms

Of particular interest is the extremely high risk of MACE in the cohort of patients with elevated plasma glucose. Indeed, levels of ≥ 11.1 mmol/L, which are considered diagnostic for diabetes in stable patients [15], were associated with a sixfold increase in mortality and a fourfold increase in MACE at 30 days in spite of current guideline-based management. Similar associations between glucose and outcomes have been seen in a small study of NSTEMI patients undergoing PCI [16], while others have also shown that hyperglycemic patients are more likely to present with STEMI [17]. Importantly, we have demonstrated that this increased risk persists up to 1 year both in the overall and landmark analysis suggesting a long-term effect beyond the acute exposure to high glucose. This is most likely explained by poor longer term glycemic control, however other mechanisms may also contribute. Indeed, glycemic variability has previously been shown to be predictive of outcomes in a cohort of patients with ACS undergoing PCI [18]. Furthermore, the risk of repeat revascularization has been shown to be associated with the severity of diabetes, with insulin dependent diabetics at highest risk of need for repeat revascularization [19].

The risk associated with markedly elevated entry glucose may at least partly reflect sympathetic stimulation. Of note, catecholamines are known to stimulate glucose release from the liver and induce hyperglycemia [20]. Heart rate, which is a crude measure of sympathetic activation, was indeed higher in patients with high rather than lower or normal entry glucose. In line with this interpretation, marked sympathetic activation in ACS patients is known to be associated with worse outcomes [21]. Accordingly, these patients also had a higher maximal creatinine kinase rise, lower ejection fraction and increased NT-BNP compared to the other two groups. Similarly, in previously published small series of patients with cardiogenic shock who do have markedly elevated catecholamines, entry glucose was also predictive [22].

Mechanistically, the higher mortality and MACE in those with high entry glucose may result from direct glucotoxic effects [23], leading to attenuated endothelium-dependent vasodilatation [24], thereby impairing the micro- and macrocirculation and and leading to reduced myocardial perfusion, ischemia and increased infarct size [25]. Hyperglycaemia can also alter platelet function and intraplatelet signalling pathways and can cause conformational changes in platelet glycoproteins [26], leading to more solid coronary clots that are associated with worse outcomes [27].

### Cyr61, STEMI and hyperglycemia

A number of novel biomarkers have been proposed in risk stratification of ACS patients. The biomarker Cyr61 is most interesting in this context as it is involved in inflammation, cell adhesion, migration and proliferation and reflects myocardial injury under ischemic conditions before troponins become elevated and predicts outcome in the ACS population [14]. In our study, we found a striking relationship between Cyr61, STEMI and hyperglycemia with no significant relationship with longer term glycemic control as measured using HbA1c or even the presence or absence of diabetes. The lack of association with long term glycemic control contrasts with previous research which demonstrated upregulation of Cyr61 in diabetics with proliferative retinopathy, a surrogate marker of diabetes severity [28]. In terms of inflammation, there was a weak negative relationship between Cyr61 and CRP, but a positive relationship between Cyr61 and leucocyte count suggesting that it is regulated by cellular immunity. Indeed, the protein is particularly highly expressed at sites of inflammation and tissue repair [29].

We have previously found Cyr61, a marker of myocardial ischemia and injury, to be elevated in STEMI patients [14], compared to NSTEMI patients. This has been replicated in another analysis that demonstrated higher Cyr61 in patients with ACS compared to stable angina and control patients without coronary artery disease [30]. Building on this, we further found Cyr61 levels in STEMI patients increased as glucose increased, whereas there was no such dose dependent effect in NSTEMI. This likely reflects the larger degree of ischaemia present in STEMI leading to elevated glucose whereas the reasons for hyperglycemia in the NSTEMI population may be more related to longer-term glycemic control and not solely to the ischemia itself. Indeed, out of 30 patients with NSTEMI and BSL ≥ 11.1 mmol/L, 29 had a diagnosis of diabetes whereas only two-thirds of STEMI patients with BSL ≥ 11.1 mmol/L had a diagnosis of diabetes.

### Obesity and inflammation

Obesity is a major risk factor for the development of diabetes [31], and in line with this observation, diabetic patients presented with a higher BMI than non-diabetic patients. The association between MACE rates and BMI showed a U-shape relationship with the highest event rate in those with a BMI of less than 25 kg/m^2^, the lowest in those with a BMI of 25 to 30 kg/m^2^ and intermediate event rate in those with a BMI of greater than 30 kg/m^2^. The increased MACE in diabetics with BMI < 25 kg/m^2^ was driven primarily by repeat revascularisation, although the 1-year risk of death was also numerically higher. Cohort studies have previously found that results similar to ours are caused by confounding and following adjustment patients with a BMI between 20 and 24.9 had a lower adjusted all-cause mortality, with excess mortality in low weight (BMI < 20 kg/m^2^) driven by extra-cardiac causes [32].

An underlying cause of the observed findings could be inflammation which is known to be associated with insulin resistance and elevated postprandial glucose levels [33]. Indeed, both diabetics and non-diabetics with elevated entry glucose also had increased levels of CRP and white blood cell count reflecting activation of both humoral and cellular inflammatory pathways. Of note, inflammation has indeed been shown to determine outcomes after ACS [34].

## Limitations

A limitation of the study is the onsite measurement of glucose in an all-comer cohort although this was always performed in arterial blood and is a highly established and standardized measurement. Also, measurement of glucose was obviously not necessarily fasting in the emergency setting, Given patients with STEMI have a shorter time from symptom onset to angiography, the blood for patients presenting with STEMI was taken earlier in their disease course compared to NSTEMI/UA patients which may confound the interpretation of some biomarkers related to ischaemia and inflammation. Furthermore, this is a registry without interventional measures allowing for definitive proof of causality of the observed findings.

## Conclusions

In this large, prospective, independently adjudicated cohort of in all comers ACS patients undergoing PCI, both a history of diabetes and most strikingly markedly elevated entry glucose > 11.1 mmol/L was associated with inflammation, elevated troponin levels and increased risk of MACE both at short and long-term. The mediators might involve increased sympathetic activation, inflammation and ischemia as reflected by elevated Cyr61 levels leading to larger infarcts and lower LVEF.

## Supplementary information

**Additional file 1: Figure S1.** Distribution of blood glucose levels in diabetics and non-diabetics in the SPUM-ACS study. **Table S1.** Demographic and major laboratory values in diabetics and non-diabetics according to blood glucose. **Table S2.** Demographics and major laboratory values in diabetics and non-diabetics according to BMI.

## Data Availability

The datasets used and/or analysed during the current study are available from the corresponding author on reasonable request.

## Abbreviations

ACS: acute coronary syndromes
BMI: body mass index
CVD: cardiovascular disease
CK_Max_: maximal plasma levels of creatinine kinase
CRP: C-reactive protein
Cyr61: cysteine-rich angiogenic inducer 61
LVEF: left ventricular ejection fraction
NTproBNP: N-terminal pro-brain natriuretic peptide
NSTEMI: non ST-segment elevation myocardial infarction
PCI: percutaneous coronary intervention
STEMI: ST-segment elevation myocardial infarction
