# Supplementary material for: Diabetes and baseline glucose are associated with inflammation, left ventricular function and short- and long-term outcome in acute coronary syndromes: role of the novel biomarker Cyr 61
Source: Cardiovasc Diabetol. 2019 Oct 31;18:142. doi: 10.1186/s12933-019-0946-6 (PMC6824030; doi:10.1186/s12933-019-0946-6)
Supplement: Supplementary file 1 — Additional file 1: Figure S1. Distribution of blood glucose levels in diabetics and non-diabetics in the SPUM-ACS study. Table S1. Demographic and major laboratory values in diabetics and non-diabetics according to blood glucose. Table S2. Demographics and major laboratory values in diabetics and non-diabetics according to BMI. [file 12933_2019_946_MOESM1_ESM.doc]

**Additional file 1**

**Figure S1:** Distribution of blood glucose levels in diabetics and non-diabetics in the SPUM-ACS study.

**
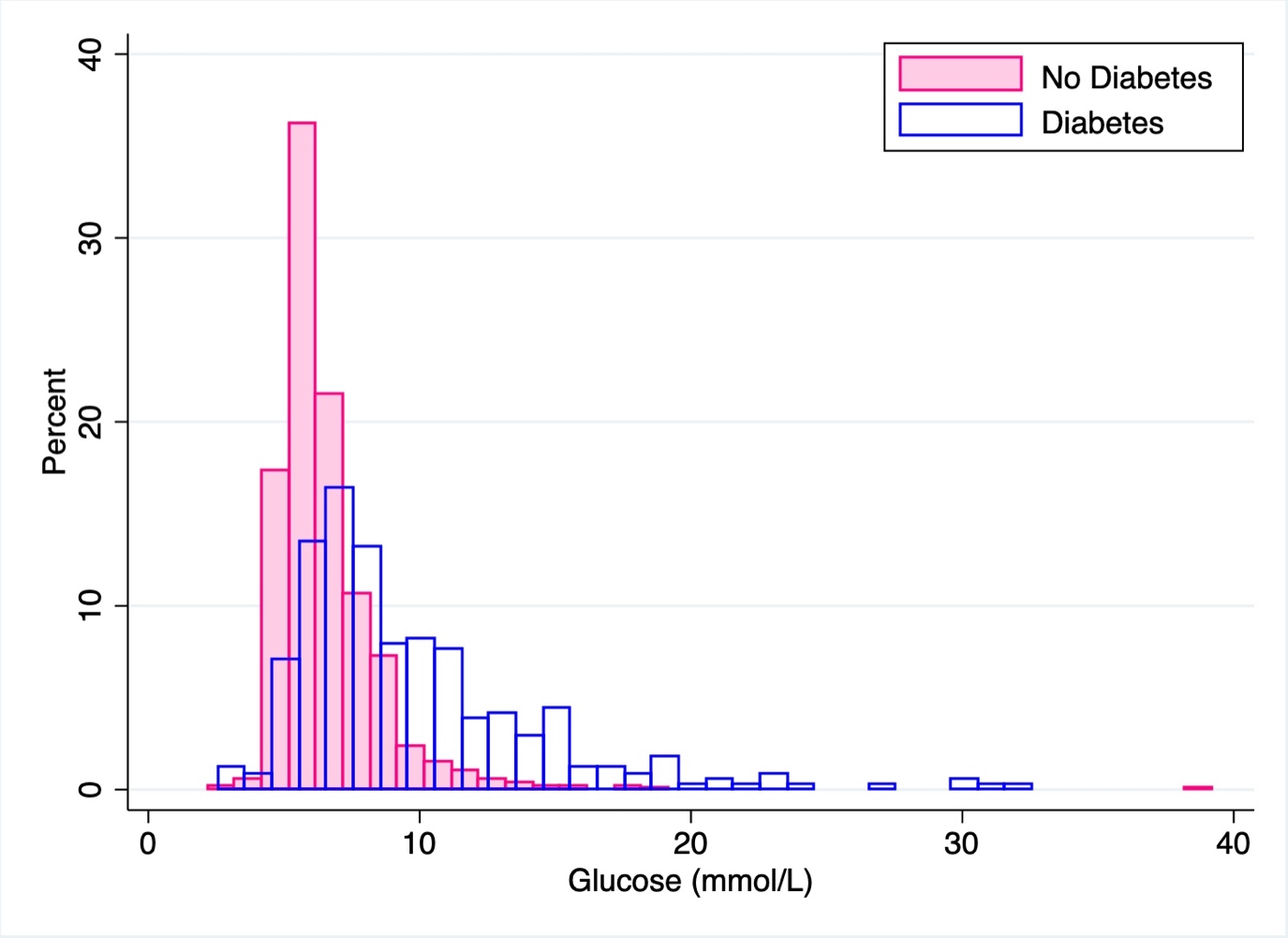
**

**Table S1:** Demographic and major laboratory values in diabetics and non-diabetics according to blood glucose

| **Variable** | **BSL < 6.0** | **BSL 6.0 - 11.1** | **BSL > 11.1** | **p-value** |
| --- | --- | --- | --- | --- |
| N | 711 | 968 | 134 |  |
| BMI | 26.8 (4.2) | 27.3 (4.4) | 28.0 (4.5) | 0.003 |
| Sex (Female) | 137 (19.3%) | 212 (21.9%) | 24 (17.9%) | 0.31 |
| (Male) | 574 (80.7%) | 756 (78.1%) | 110 (82.1%) |  |
| Age | 62.9 (12.5) | 63.6 (12.1) | 65.6 (13.2) | 0.068 |
| HDL | 1.2 (0.3) | 1.2 (0.3) | 1.1 (0.3) | 0.032 |
| LDL | 3.1 (1.1) | 3.2 (1.2) | 3.0 (1.2) | 0.010 |
| triglyceride | 1.05 (0.69-1.52) | 1 (0.66-1.58 | 1.12 (0.74-1.81) | <0.001 |
| Total cholesterol | 4.9 (1.2) | 5.0 (1.3) | 4.9 (1.4) | 0.034 |
| LVEF | 55 (45-60) | 50 (42-60) | 45 (39-59.5) | <0.001 |
| HR | 74.6 (15.4) | 76.7 (16.3) | 80.6 (17.5) | <0.001 |
| SBP | 131.3 (23.2) | 129.7 (22.9) | 130.2 (25.8) | 0.42 |
| DBP | 75.2 (14.3) | 76.1 (14.6) | 76.1 (18.1) | 0.41 |
| Leucocyte count | 8.7 (6.9-11.1 | 10.2 (8.1-12.9) | 11.3 (9.1-14.5) | <0.001 |
| CRP | 2.7 (1.1-7.6 | 2.7 (1.2-7.4) | 3.5 (1.5-11.4) | 0.035 |
| glucose | 5.3 (0.5) | 7.4 (1.2) | 15.0 (4.7) | <0.001 |
| HbA1c | 5.9 (0.8) | 6.4 (1.2) | 8.0 (2.5) | <0.001 |
| hsTnT, | 0.19 (0.06-0.61) | 0.20 (0.06-0.73) | 0.28 (0.09-1.11) | 0.13 |
| CK | 209 (103-428) | 241 (114-550) | 248 (109-617) | 0.001 |
| NT-proBNP | 371 (129-1260) | 337 (106-1199) | 520 (142-2036) | 0.009 |
| Cyr61 | 405 (299-615) | 611 (382-1122) | 656 (381-1624) | <0.001 |

**Table S2:** Demographics and major laboratory values in diabetics and non-diabetics according to BMI.

| **Variable** | **<25kg/m^2^** | **25-29.9kg/m^2^** | **>30kg/m^2^** | **p-value** |
| --- | --- | --- | --- | --- |
|  | N = 641 | N = 922 | N = 441 |  |
| BMI | 22.8 (1.7) | 27.1 (1.3) | 33.4 (3.3) | <0.001 |
| Sex (Female) | 180 (28.1%) | 151 (16.4%) | 86 (19.5%) | <0.001 |
| Sex (Male) | 461 (71.9%) | 771 (83.6%) | 355 (80.5%) |  |
| Age | 65.6 (12.6) | 63.2 (12.2) | 60.7 (11.9) | <0.001 |
| HDL (mmol/L) | 1.3 (0.4) | 1.1 (0.3) | 1.1 (0.3) | <0.001 |
| LDL (mmol/L) | 3.1 (1.2) | 3.3 (1.1) | 3.2 (1.1) | 0.006 |
| Triglyceride (mmol/L) | 0.85 (0.58-1.22) | 1.1 (0.72-1.65) | 1.27 (0.87-2.03) | <0.001 |
| Cholesterol (mmol/L) | 4.8 (1.3) | 5.0 (1.3) | 5.0 (1.3) | 0.009 |
| LVEF (%) | 50 (44-60) | 52 (45-60) | 50 (45-60) | 0.91 |
| HR (BPM) | 76.8 (16.9) | 74.8 (15.4) | 77.4 (15.2) | 0.005 |
| Systolic BP (mmHg) | 128.6 (23.2) | 130.2 (23.4) | 133.9 (23.2) | 0.001 |
| Diastolic BP (mmHg) | 73.9 (14.7) | 75.9 (14.7) | 78.2 (15.1) | <0.001 |
| Leucocyte count | 9.8 (7.7-12.4) | 9.6 (7.4-12.1) | 9.6 (7.7-12.3) | 0.43 |
| CRP (mg/L) | 2.3 (0.9-6.6) | 2.5 (1.1-7.0) | 3.9 (1.7-10.1) | 0.075 |
| Glucose (mmol/L) | 6.9 (2.6) | 7.1 (2.9) | 7.5 (3.1) | 0.005 |
| HbA1c (%) | 6.3 (1.1) | 6.6 (1.5) | 7.0 (2.3) | 0.005 |
| hsTnT | 0.23 (0.06-0.76) | 0.20 (0.06-0.69) | 0.17 (0.06-0.65) | 0.45 |
| CK | 214 (106-492 | 228 (107-521) | 232 (112-507) | 0.94 |
| NT-pro-BNP | 479 (152-1577) | 348 (112-1199) | 320 (104-1157) | <0.001 |
| Cyr61 | 570 (367-1117) | 485 (331-879) | 428 (324-727) | 0.018 |
